# Minimising oxygen contamination through a liquid copper-aided group IV metal production process

**DOI:** 10.1038/s41598-018-35739-z

**Published:** 2018-11-26

**Authors:** Bung Uk Yoo, Young Jun Lee, Vladislav Ri, Seong Hun Lee, Hayk Nersisyan, Hyun You Kim, Jong Hyeon Lee, Nicholas Earner, Alister MacDonald

**Affiliations:** 1 Graduate school of Energy Science and Technology, Chungnam National University, 99 Daehak-ro, Daejeon, 34134 Republic of Korea; 2 Rapidly Solidified Materials Research Center (RASOM), Chungnam National University, 99 Daehak-ro, Daejeon, 34134 Republic of Korea; 3 ZIRON Tech Co. Chungnam National University, 99 Daehak-ro, Daejeon, 34134 Republic of Korea; 4 Department of Materials Science and Engineering, Chungnam National University, 99 Daehak-ro, Daejeon, 34134 Republic of Korea; 5 Alkane Resources Ltd, 89 Burswood Road, Perth, Western Australia 6100 Australia

## Abstract

This paper demonstrates for the first time the fabrication of Zr-Cu alloy ingots from a Hf- free ZrO_2_ precursor in a molten CaCl_2_ medium to recover nuclear-grade Zr. The reduction of ZrO_2_ in the presence of CaO was accelerated by the formation of Ca metal in the intermediate stage of the process. Tests conducted with various amounts of ZrO_2_ indicate that the ZrO_2_ was reduced to the metallic form at low potentials applied at the cathode, and the main part of the zirconium was converted to a CuZr alloy with a different composition. The maximum oxygen content values in the CuZr alloy and Zr samples upon using liquid Cu were less than 300 and 891 ppm, respectively. However, Al contamination was observed in the CuZr during the electroreduction process. In order to solve the Al contamination problem, the fabrication process of CuZr was performed using the metallothermic reduction process, and the produced CuZr was used for electrorefining. The CuZr alloy was further purified by a molten salt electrorefining process to recover pure nuclear-grade Zr in a LiF-Ba_2_ZrF_8_-based molten salt, the latter of which was fabricated from a waste pickling acid of a Zr clad tube. After the electrorefining process, the recovered Zr metal was fabricated into nuclear-grade Zr buttons through arc melting following a salt distillation process. The results suggest that the removal of oxygen from the reduction product is a key reason for the use of a liquid CaCu reduction agent.

## Introduction

Direct reduction could significantly simplify the isolation of group IV transition metals from their corresponding oxides (for example TiO_2_, ZrO_2_, and HfO_2_). These metals are typically produced by the Kroll process^1,2^ but the multiple stages, especially the chlorination step, reduces the effectiveness of the extraction, and increases the cost of the final product and the environmental impact. Metal oxides have been directly reduced to their corresponding metals by electrochemical^3–12^ and metallothermic approaches^13,14^. However, the conventional electrochemical reduction only achieves partial oxygen removal^7–9^. This is a serious disadvantage for producing ductile transition metals on a commercial scale. The electrochemical method is inadequate for nuclear-grade Zr production because the metal loses its ductility under loss-of-coolant nuclear reactor accident conditions in the presence of impurities, especially for oxygen contamination exceeding 1400 ppm^15,16^. Here, we report a metal production technology that lowers the oxygen content of Zr to meet the requirements for nuclear-grade metal. During this process, the target metal is kept mixed with another metal, in its liquid state, which shields it from CaO-containing electrolytes. We demonstrate the indirect electro-fabrication of CuZr alloy ingots from a low-Hf ZrO_2_ feedstock in molten CaCl_2_ using a liquid Cu cathode (LCC). Once the CuZr alloy ingot is prepared, pure Zr can be readily obtained from electrorefining without the chlorination process. Density functional theory (DFT) molecular dynamics (MD) simulations provided details about the key processes leading to CuZr formation after ZrO_2_ reduction. As the oxide-containing Zr as a solid solution did not form a CuZr phase due to repulsive forces, only oxygen-free Zr is expected in the Cu. The combined theory–experiment study suggests that this ZrO_2_ reduction method can be generalised to the simple reduction of metal oxides.

## Results and Discussion

**Figure 1:**
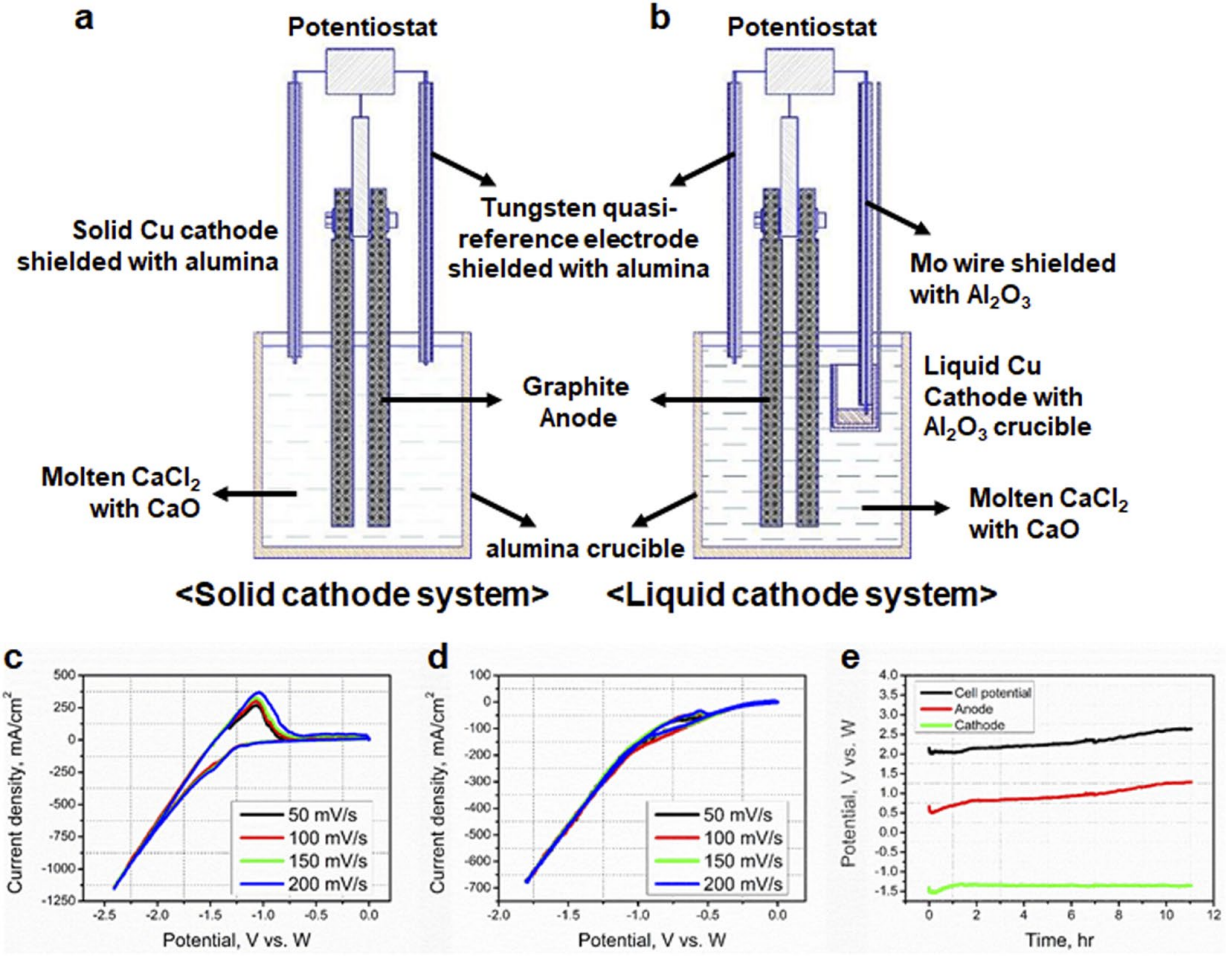
Electroreduction of ZrO_2_ in a CaCl_2_–CaO molten salt. (**a**) Solid copper cathode system at 1080 K. (**b**) CVs of the solid cathode system at various scan rates. (**c**) CV results of the liquid cathode system at various scan rates. (**d**) Liquid copper cathode system at 1380 K. (**e**) Chronopotentiometry during electroreduction at E4 experimental conditions in CaCl_2_ with 5 wt% CaO at 1380 K. The E4 condition means that 8.47 g of ZrO_2_ is charged on the 10 g of liquid copper and a current of 1A is applied for 11.054 hours. The weight ratio of ZrO_2_/Cu is 84.7.

The production of nuclear-grade Zr by chlorination-free method starts from a preparation of CuZr ingot that was initially obtained by ZrO_2_ through electroreduction or metallothermic using liquid Cu as the cathode. The resulting CuZr is used for the electrorefining process in the second step. Figure 1a and c show the electrochemical cell using solid and liquid copper cathodes, respectively. Cyclic voltammograms (CVs) were obtained in the CaCl_2_ + 5 wt% CaO electrochemical system at 1080 K vs. the W reference electrode (Fig. 1b). The process temperature was lower than the melting point of Cu (T_melt._ = 1358 K), and therefore, the Cu cathode was in the solid state. The Ca^2+^ reduction process on the Cu solid cathode can be written as:

1 $${{\rm{Ca}}}^{2+}\,({\rm{from}}\,{\rm{CaO}})+2{{\rm{e}}}^{-}\to {\rm{Ca}}^\circ \,({\rm{E}}=-\,1.25\,{\rm{V}})$$

The reduction potential of the CaCl_2_ + 5 wt% CaO system increased to −0.8 V at 1380 K (above the melting point of Cu) (Fig. 1d). Two main reasons for the change in the reduction potential are related to the process temperature and the liquid electrode. Therefore, an applied potential of at least −1.5 V is needed to effectively reduce ZrO_2_, and this value can be reached by applying a current density of 500 mA/cm^2^ to the cathode (see Fig. S2).

According to experimental observations, the part of the Cu cathode that was immersed in the molten chloride melted, despite the system temperature being below the melting point of Cu due to intermetallic compound between Cu and Ca decrease the melting point. We assumed that this melting of the Cu(Ca) cathode provoked a sharp change in the reduction potential due to formation of an intermetallic compound between Cu and Ca^17^. The CVs (Fig. 1c and d) displayed minimal oxidation peaks because the reduced Ca metal immediately reacted with the Cu electrode to produce a CaCu alloy. This phenomenon is noticeable in liquid Cu cathodes because the diffusion of reduced Ca on a liquid rather than a solid Cu cathode leads to rapid redistribution inside the Cu. The reduction potential change resulted from the temperature difference and the underpotential deposition phenomenon due to negative Gibbs formation energy of CaCu intermetallic compound, which allowed the reduced Ca to react more easily with the liquid Cu cathode than its solid analogue (see Fig. S2). Thus, the CV tests confirmed the formation of Ca metal in both solid and liquid cathode systems. Through this experiment, it was confirmed that a reducing agent suitable for the reduction of ZrO_2_ can be prepared electrochemically because it is heavier than the electrolyte.

The electrochemically produced CaCu is used simultaneously for the reduction of ZrO_2_. The reduction of ZrO_2_ was monitored by chronopotentiometry at an applied current density of 500 mA/cm^2^ (Fig. 1e). Chronopotentiometry measurements for various ZrO_2_ concentrations (E1;6.2, E2;15.2, E3;40.1 and E4;84.7 in ZrO_2_/Cu mass ratio) are detailed in the Methods Section. The cell potential was defined as the potential difference between the cathode and anode. The cathode potential presented a negligible increase from −1.41 to −1.35 V vs. W over 11 h at an applied current density of 500 mA/cm^2^. In contrast, the graphite anode potential increased from 0.62 to 1.29 V. This change can be explained by a decrease in the anode surface area during the electroreduction^18^. In the case of an electroreduction in which solid matter exists before and after the reaction, such as Fray-Farthing-Chen (FFC) Cambridge process and Ono-Suzuki (OS) process, the oxygen diffusion becomes difficult as the reaction progresses, and the cathode potential tends to become more negative. Therefore, the stable cathode potential in this experiment indicates that the rate of reduction dramatically increases through rapid diffusion of electrodeposits resulting from the use of a liquid cathode. This method of recovering reduced Zr as a liquid phase is considered as one of the most important requirements in the commercialisation of the electroreduction process.

The ZrO_2_ reduction process in a liquid phase can be expressed through the following equations.

2 $${\rm{Ca}}+{\rm{xCu}}={{\rm{CaCu}}}_{{\rm{x}}}\,({\rm{liquid}})$$

3 $$2{{\rm{CaCu}}}_{{\rm{x}}}\,({\rm{liquid}})+{{\rm{ZrO}}}_{2}\,({\rm{solid}})={{\rm{Cu}}}_{2{\rm{x}}}{\rm{Zr}}\,({\rm{liquid}})+2{\rm{CaO}}$$

**Figure 2:**
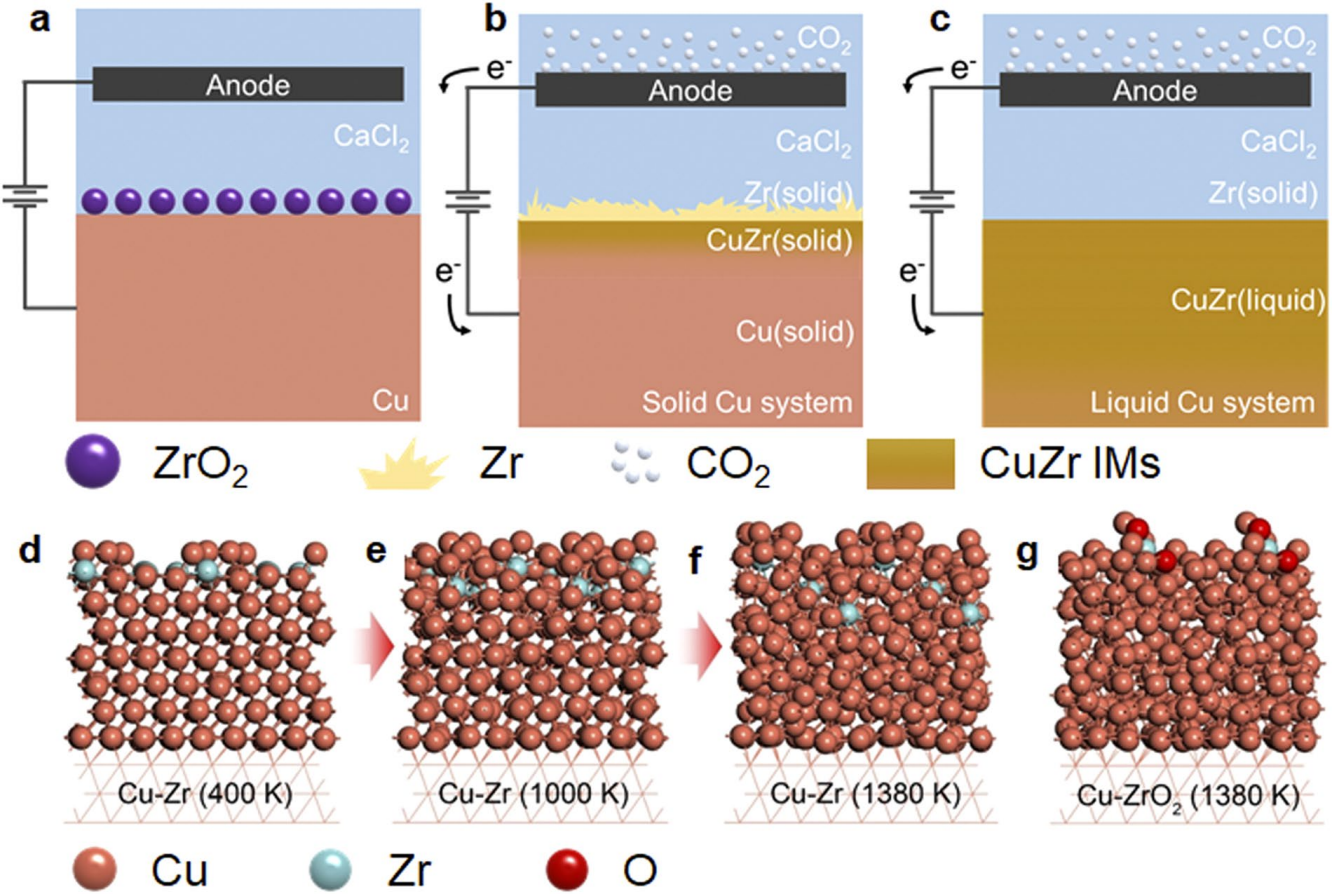
Reduction behaviour of ZrO_2_ according to the process temperature. Schematic representations of the electroreduction system at (**a**) the initial state, (**b**) below the melting point of Cu, and (**c**) above the melting point of Cu. DFT-MD simulation snapshots after 10 ps of reaction time at (**d**) 400 K, (**e**) 1000 K, and (**f**) 1380 K. (**g**) The simulations indicated that ZrO_2_ on the Cu(111) surface at 1380 K did not penetrate into liquid Cu phase because of repulsive forces. Copper, cyan, and red spheres represent Cu, Zr, and O atoms, respectively.

For the understanding of the mechanism of ZrO_2_ reduction and CuZr liquid phase formation, a schematic diagram is presented in the Fig. 2a–c. Because ZrO_2_ powder exhibits a lower density than liquid or solid Cu, it is always located on the Cu cathode surface. As a result, it accumulates on the Cu cathode when added to the electrochemical cell (Fig. 2a). Below the Cu melting point, most of the reduced Zr accumulates on the Cu cathode surface while a small portion diffuses into the cathode (Fig. 2b). The extremely large surface area of the solid particles of the Zr product hinders the complete removal of oxygen from ZrO_2_ when exposed to the CaO-enriched molten salt due to the chemical equilibrium of Ca/CaO^19^. However, above the melting point of Cu, the oxygen concentration in the product can be greatly reduced because Zr reacts with Cu to form intermetallic compounds during the reduction process. This reaction prevents the re-oxidation of Zr and any side reaction with CO_2_ generated from the anode (Fig. 2c). Moreover, because the liquid CuZr phase is sufficiently denser than that of CaCl_2_, phase separation spontaneously occurs, and washing the product with water to remove electrolytes is not required; hence, re-oxidation of Zr during post-treatment can be essentially prevented.

The formation of a CuZr alloy at high temperatures was assessed by DFT-MD simulations. Figure 2f shows that ZrO_2_ reduced by Ca penetrated into the Cu(111) substrate at 1380 K, which was not observed at lower temperatures (Fig. 2d, 400 K and Fig. 2e, 1000 K). The Zr–Zr cohesive energy was greater than its Cu–Cu equivalent and the Cu–Zr interaction energy lay between these energies^20^, meaning that the formation of CuZr alloy was thermodynamically endothermic. The Cu–Zr alloying limit occurs above the melting point of Cu. Consequently, melting-induced structural disorder would provide room for additional Cu–Zr bonds and initiate Zr penetration into Cu.

Supplementary DFT-MD simulations also showed that a Zr cluster containing oxygen as the feedstock in the electroreduction process that was initially supported on a Cu(111) substrate could not penetrate the liquid Cu (see Fig. 2g) even as the temperature rises to 1380 K. The Zr that contains oxygen as a solid solution did not form a CuZr phase due to repulsive forces. Thus, a significantly low oxygen concentration of less than 300 ppm in Zr was expected in the Cu as observed in the oxygen analysis data.

Metal reduction by the conventional metallothermic process is determined by^19^:

4 $${\rm{O}}\,({\rm{in}}\,{\rm{Ti}})+{\rm{Ca}}\,({\rm{in}}\,{\rm{salt}})={\rm{CaO}}\,({\rm{in}}\,{\rm{salt}})$$

5 $$[ \% {\rm{O}}]=({a}_{CaO}/{a}_{Ca})1/{f}_{0})\,\exp ({\rm{\Delta }}{G}^{0}/RT)$$

where Δ*G*^0^ is the standard free energy change of the reaction (Eq. (5)), *a*_*CaO*_ and *a*_*Ca*_ are the activities of CaO and calcium, respectively, and *f*_0_ is the activity coefficient of oxygen in solid titanium. Because the oxygen content is determined by the thermodynamic properties as long as the solid metal product is in contact with the salt, existing processes having a large reaction surface area are limited in reducing the oxygen content. Therefore, a lower oxygen content in the metal rests on decreasing in CaO activity in the electrolyte or increasing the Ca activity. The key deoxidation steps of the liquid Cu-assisted reduction are indirect reduction and intermetallic compound formation, which produces the liquid CuZr alloy, simultaneously with the Ca-mediated metallothermic reduction of ZrO_2_. In addition, by separating the CaO-containing electrolyte from the liquid CuZr phase based on the specific gravity difference, the CaO is not subject to the chemical equilibrium reaction mentioned in Eqs (2) and (3). In the present process, the chemical equilibrium reaction can occur at the interface between the salt and CuZr phase, but, as suggested by the above-mentioned DFT-MD simulations, oxygen-bound Zr cannot penetrate into the Cu. Therefore, an extremely low oxygen content can be achieved.

**Figure 3:**
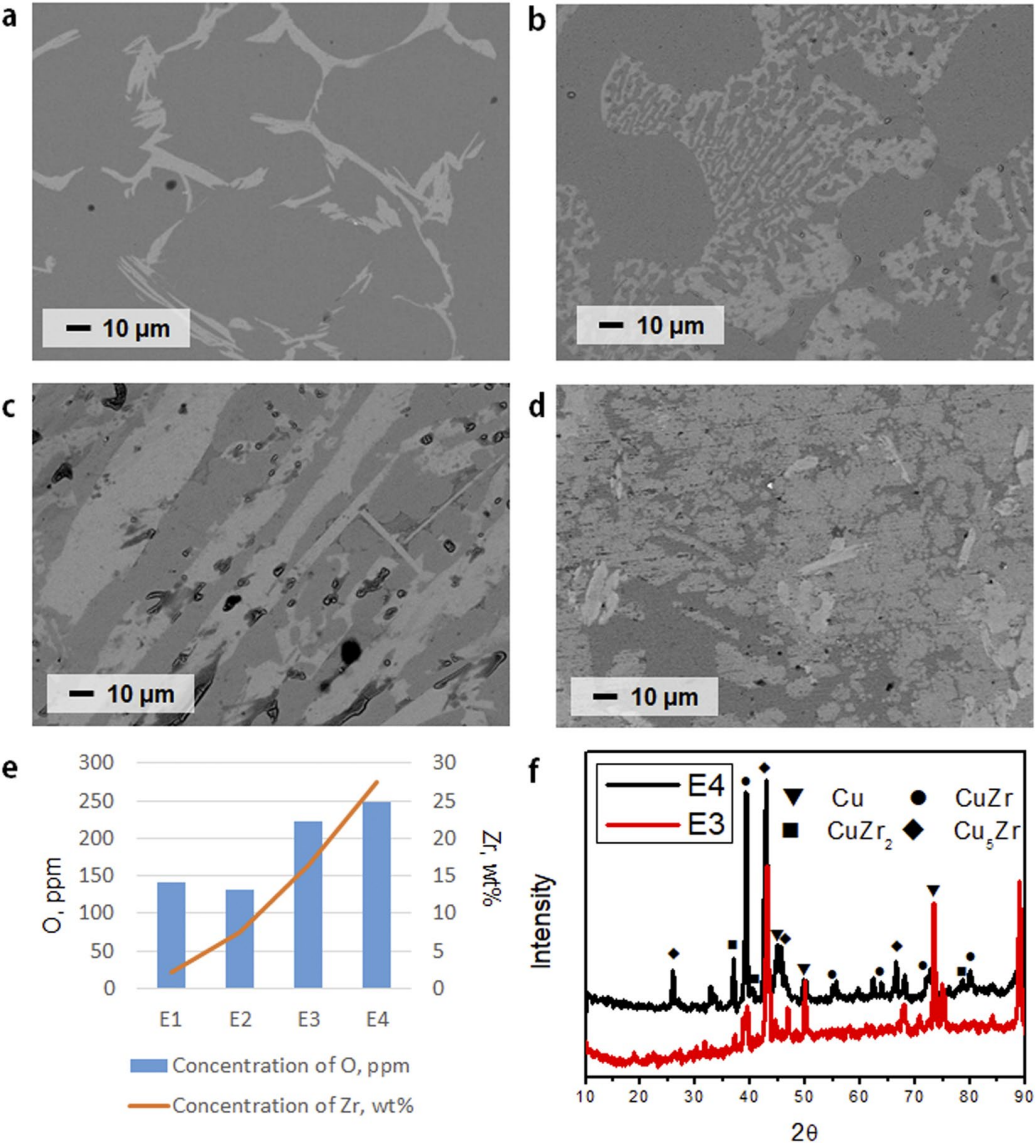
The microstructures displayed CuZr (white, **a**–**d**) as well as CuAl intermetallic compounds (dark gray, **a**–**d**). The CuAl portion decreased with increasing ZrO_2_ concentration (**d**) for the benefit of the CuZr alloy. (**a**) E1;6.2, (**b**) E2;15.2, (**c**) E3;40.1, and (**d**) E4;84.7 in ZrO_2_/Cu mass ratio. Under each condition, a current of 1A was applied to 10 g of liquid copper. (**e**) Zr and O concentrations in the cathode ingots. (**f**) Comparison between XRD patterns for different CuZr samples.

The energy dispersive X-ray (EDX) analysis of the recovered CuZr detected Cu, Zr, and Al elements but no oxygen in the samples (see Table S3). The presence of Al originated from the Al_2_O_3_ crucible during the reduction process. The microstructures of CuZr alloys obtained under various electroreduction conditions were analysed by back-scattered electron imaging (×500) in which heavy elements (Zr in this analysis) back-scatter electrons more strongly than light elements (Cu and Al in this analysis), and thus appear brighter in the image (Fig. 3a–d). With increasing Zr concentrations, the shape of the grains became columnar, which is typical for alloy systems^21,22^. Also, a refinement of the grains was noticeable above a certain Zr concentration (>30 wt%, Fig. 3d). This may result from the simultaneous formation of several local crystallisation centres in the alloy sample. The oxygen content values of samples E1–E4 (Fig. 3e) analysed by Eltra ONH-2000 ranged from 142 to 249 ppm, which are acceptable levels for nuclear-grade Zr^23^.

Through quantitative analysis, it was confirmed that the reduced Zr concentration increases as the amount of ZrO_2_ is increased. Then, XRD pattern analysis was performed to observe the phase change of CuZr formed as the amount of reduced Zr increased. The main phases detected in alloy E3 (40.1, ZrO_2_/Cu weight ratio) were Cu, Cu_5_Zr, and CuZr (Fig. 3f). When the Zr content increased to 28 wt%, an alloy phase corresponding to CuZr_2_ appeared in the reaction product (Fig. 3f). The amount of the CuZr phase also increased sufficiently. Based on the XRD data, it is difficult to determine the sequence of formation of the different alloy phases during the electroreduction. Aluminum contamination from the Al_2_O_3_ crucible was observed in CuZr production by electroreduction. Therefore, ZrO_2_ reduction was carried out by a metallothermic method using CaCu to prevent Al pollution. As a result, it was possible to recover the Al-free CuZr ingot in quantities suitable for industrial use (see SI for details, Figs S3–S8). Electroreduction and CaCu-mediated metallothermic reduction can effectively remove oxygen from low-Hf ZrO_2_. The presence of CaCu alloy in these processes can produce a large amount of CuZr alloy. It is possible to prevent co-reduction of the Al_2_O_3_ crucible by using CaCu as a reductant and insoluble metallic crucibles such as those made of Mo or W. This CuZr ingot was used as anode feedstock to obtain Zr.

**Figure 4:**
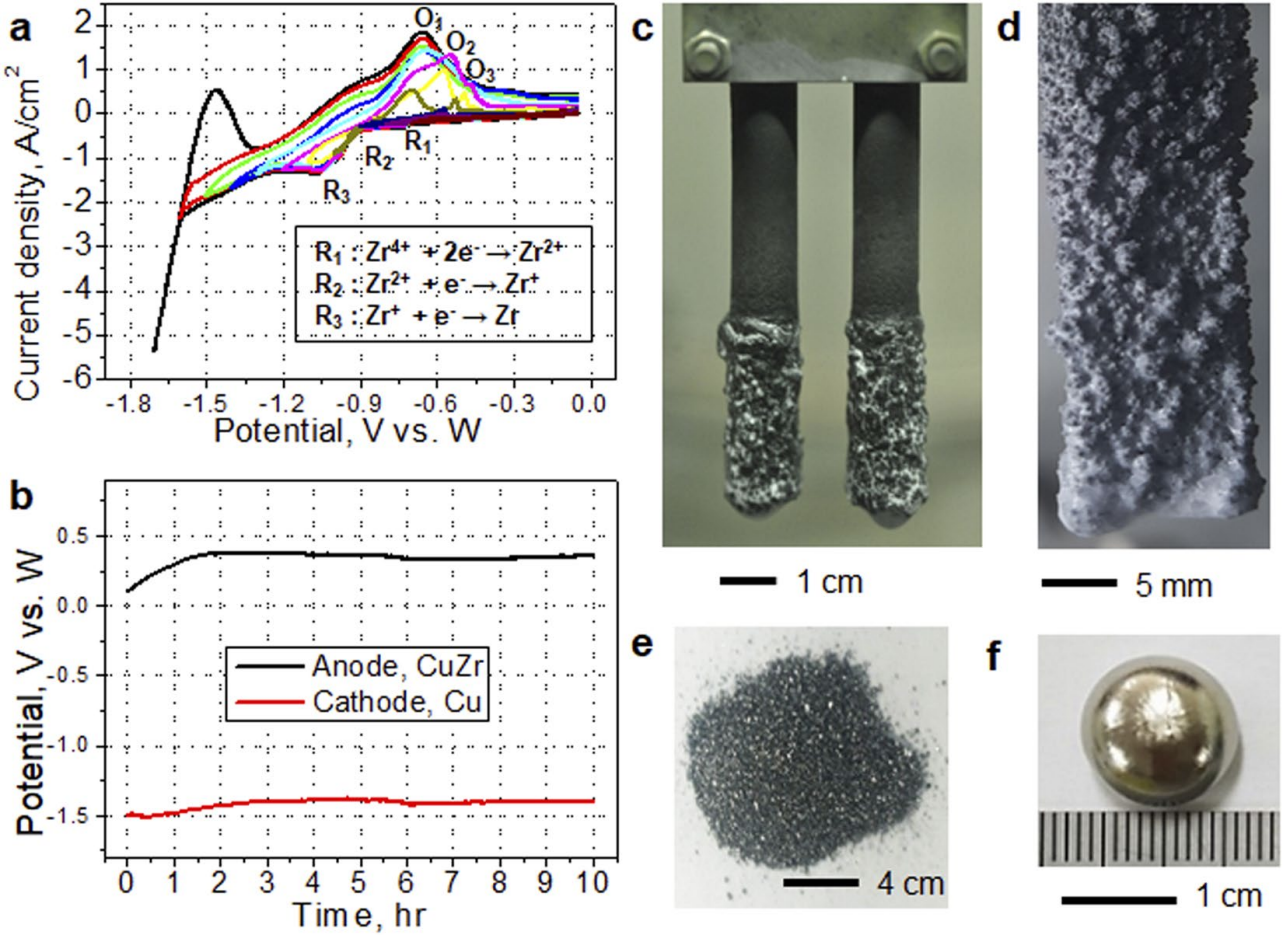
Electrorefining of Zr using a LiF–Ba_2_ZrF_8_ molten salt system. (**a**) Cyclic voltammograms obtained under various scan range conditions at 1053 K. (**b**) Chronopotentiometry during the electrorefining process at 1053 K. Photographs of the (**c**) anode and (**d**) cathode after 10 h of electrorefining. (**e**) Deposition of Zr on the cathode after vacuum distillation. Photographs of the nuclear grade Zr powder after vacuum distillation process of cathode electrodeposits. (**f**) Nuclear-grade Zr button.

A low-Hf ZrF_4_-containing electrolyte, such as Ba_2_ZrF_8_, was needed to recover pure Zr from the CuZr ingot by electrorefining. An economic way to secure such electrolytes from waste pickling acid has already been reported in our previous research^24^. In order to produce nuclear grade Zr, it is necessary to secure the electrochemical potential condition capable of recovering pure Zr by selectively dissolving Zr from the CuZr alloy ingot through the electrorefining. The behaviour of the Zr^4+^ ion in the LiF–Ba_2_ZrF_8_ molten salt system was evaluated by CV (Fig. 4a). The reduction of Zr^4+^ ion consists of a 3-step electrorefining process. The number of reactive electrons was confirmed by Eq. (6)^25,26^ using each reduction potential peak in Fig. 4a.

6 $$|{{E}}_{{p}}-{{E}}_{p{/}2}|={0.774}({RT}{/}{nF})$$

In Eq. (6), *Ep* is the reduction potential peak and *E*_*p/2*_ is half of the Ep. Electrode potentials were stable during the electrorefining process. During this process, the Cu remained on the anode while the Zr dissolved before depositing with the salt on the cathode. The anode behaviour is detailed in the Supplementary Information. The anode and cathode after electrorefining are shown in Fig. 4c and d, respectively. Zirconium was recovered as a powder using vacuum distillation (see Fig. 4e) and nuclear-grade Zr metal buttons were subsequently generated by arc melting. Analysis of the resulting Zr metal button showed that the concentration of major impurities was very low, and contamination of molybdenum, aluminium, copper, etc., which could be contaminated from the crucible and the anode, was satisfactorily blocked. In addition, the contents of oxygen and nitrogen were 891 and 10 ppm respectively, which proved that this process is very effective for preventing pollution of gas impurities. Theoretically, oxygen contamination should be smaller than the above values, but oxygen contamination may have resulted from atmospheric exposure during transportation between the unit processes: the salt distillation and ingot manufacturing processes after electrorefining. The purity of the recovered metal satisfied the ASTM B349 specifications for nuclear-grade Zr (Table S2)^23^.

**Figure 5:**
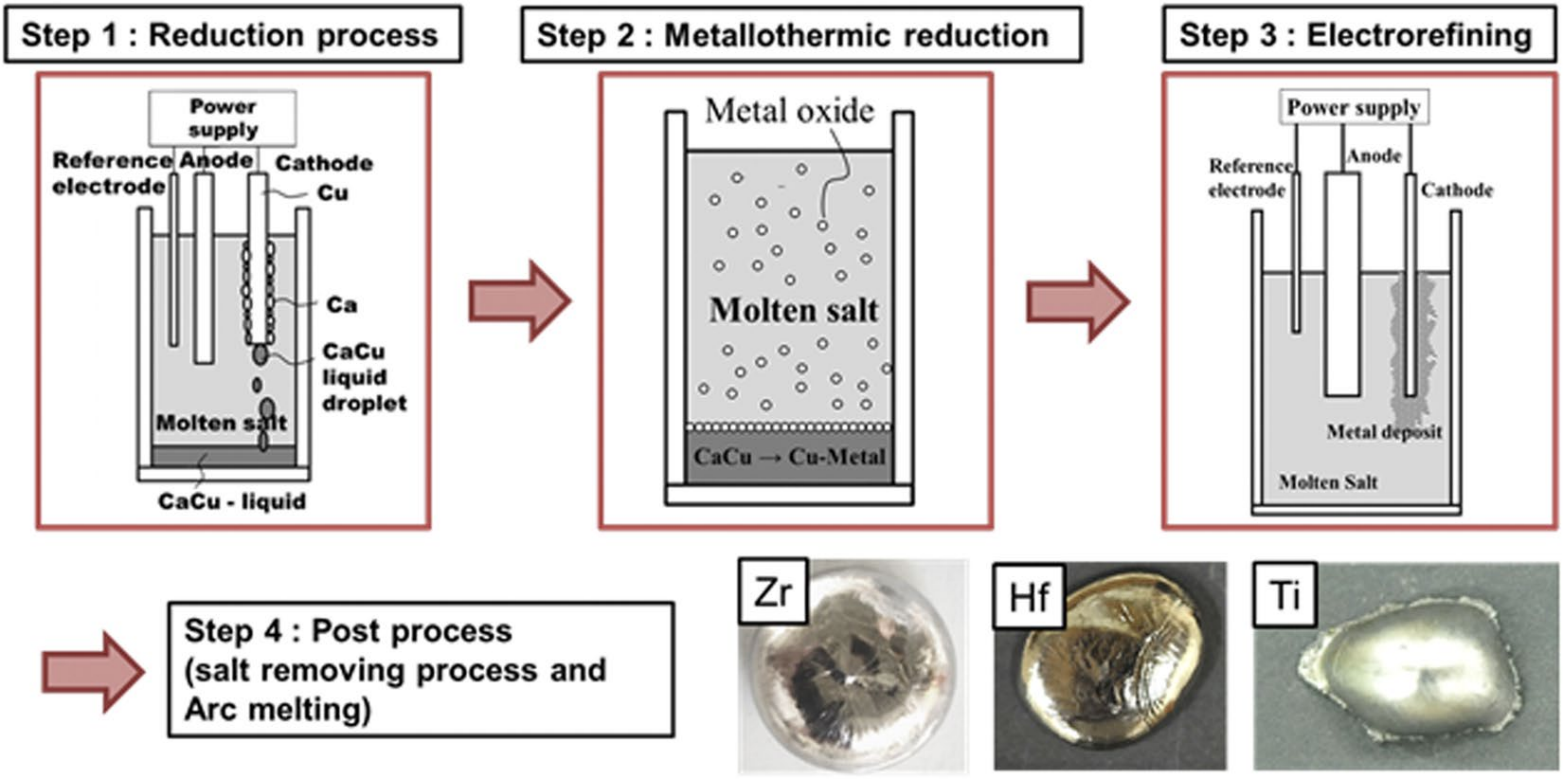
A graphical abstract showing the steps of each process to recover group IV transition metals, such as Zr, Hf and Ti.

In summary, a CuZr alloy ingot was prepared from a low-Hf ZrO_2_ precursor in a CaCl_2_–CaO molten salt using an LCC at a temperature of 1380 K (Fig. 5). The ingot exhibited an extremely low oxygen concentration. Zirconium metal was isolated from a CuZr ingot produced by metallothermic reduction via an electrorefining process in Ba_2_ZrF_8_-based electrolyte derived from waste pickling acid. Despite the fact that the metal was produced by a direct reduction process from the oxide without the chlorination process, the prepared nuclear-grade Zr displayed superior quality. In addition to producing nuclear-grade Zr, this technology can be applied to the preparation of group IV transition metals, such as Ti and Hf. And this process could be an attractive alternative to the Kroll process by avoiding environmental problems.

## Method

### Materials and equipment

Anhydrous CaCl_2_ powder (purity 98%), CaO powder (purity 99%), and low-Hf ZrO_2_ (impurities are listed in Table S2) were supplied by Samchun Chemicals (Korea), Chemicals (Japan) and Alkane Resources (Australia), respectively. The process flowsheet for the Dubbo Project, Australia, which is a new source of zirconium, hafnium, rare earth metals, and niobium, consisted of a sulphuric acid leaching of a polymetallic orebody, followed by solvent-extraction recovery and refining. Low-Hf ZrO_2_ was produced by a proprietary process to remove hafnium from a high-purity zirconium stream.

Copper chips (purity: 99.9%) and wire (1 mm, purity: 99.9%) were obtained from Junsei Chemicals (Japan) and Alfa Acer (USA), respectively. A CaCl_2_ electrolyte was used, and CaO was added for the electroreduction process. Ba_2_ZrF_8_ was synthesised using BaF_2_ (Alfa Aesar, purity >90%) and an acidic waste solution (H_2_O:HF:HNO_3_:Zr = 84:1.2:14.8:1.3 wt%) from KNF (Korea Nuclear Fuel). The electrorefining electrolyte was prepared using 65 mol% LiF (Alfa Aesar, purity >99.9%) and 35 mol% Ba_2_ZrF_8_. All raw materials were preheated at 623 K for 24 h to remove residual moisture.

Tungsten metal wires and graphite rods (diameter:1 mm) used as electrodes were 99.9% pure, as supplied by Sigma Aldrich (USA) and Shin Sung Carbon (Korea) companies, respectively. Al_2_O_3_ crucibles and tubes were supplied by Mesto, Korea. Every experiment was performed in a glove box with a stainless-steel container constructed to prevent oxidation of the electrolyte components and structural materials. The glove box was operated in an argon atmosphere in which the concentration of oxygen and moisture were controlled to be less than 2 ppm. Electrochemical measurements and electrolysis were performed using an Autolab model PGSTAT302N and NOVA computer software.

### Electrochemical procedures

#### Electroreduction

The electrochemical behaviour of Ca^2+^ ions in the electrolyte was evaluated by CV using solid and liquid Cu cathodes and a graphite anode. Components of the experimental apparatus are shown in Fig. S1. A Cu wire was used as a solid cathode while a chip provided the liquid electrode. Cathode and anode potentials were monitored using a W wire as a pseudo-reference electrode. The electrolytic system was kept at 1080 and 1380 K during the CV tests to obtain solid and liquid Cu cathodes, respectively.

Figure 1a and b show the electrochemical cell used for electrolytic reduction tests on ZrO_2_. During these tests, CaCl_2_ (1.25 kg) and CaO (62.5 g) were melted in an Al_2_O_3_ crucible with a 100-mm inner diameter placed in a stainless steel vessel and heated externally using an electric furnace. The temperature was maintained at 1380 ± 10 K. The graphite anode, W reference electrode, and liquid Cu cathode were immersed in the salt a type-K thermocouple sheathed in an Al_2_O_3_ tube. To prepare the liquid cathode, copper chips (10 g) and ZrO_2_ powder (0.62–8.47 g) were loaded into a small Al_2_O_3_ crucible, which was held in the electrolyte for 30 min. An additional W wire (1.0-mm diameter) acting as a conductor was inserted into the small crucible. Experimental conditions are summarised in Table S1. After the electroreduction, part of the cathode product was removed from the Al_2_O_3_ crucible and the electrolyte was washed off with distilled water.

#### Electrorefining

The reduction potential of Zr at 1053 K was determined by CV using the binary electrolyte LiF–Ba_2_ZrF_8_ (35:65, mol%). A Mo wire, W rod, and W wire electrode were used as the cathode, anode, and reference electrode, respectively. The electrorefining of Zr from a CuZr ingot resulting from metallothermic reduction was performed by chronopotentiometry using CaCu as a reductant. In this experiment, CuZr ingot, a Cu plate, and a W wire electrode were used as the anode, cathode, and reference electrode, respectively.

#### Post-electrorefining treatment

The electrodeposited Zr was ground in a globe box with an Ar gas atmosphere and the electrolyte was effectively removed by salt distillation for 24 h at 1573 K under vacuum (pressure: 10^−2^ Torr). Metal powders were recovered in the glove box (Fig. 4e) and Zr buttons (Fig. 4f) were prepared by arc melting under vacuum (10^−5^ Torr).

### Material characterization

Isolated materials, such as CuZr and Zr, were characterised by X-ray (CuKα radiation) diffraction (XRD, D/MAX-2200) and field-emission scanning electron microscopy (FE-SEM, JEOL JSM-6700F) in combination with energy dispersive X-ray analysis (EDX). The residual oxygen and nitrogen in the CuZr alloy and produced Zr ingot were quantified using an Eltra ONH-2000 analyser (Germany), and the low-Hf ZrO_2_ and produced Zr ingot were analysed using a glow discharge mass spectrometer (GD-MS, Msi GD90RF, UK).

#### DFT-MD simulations

DFT-MD simulations were performed using the Vienna *ab initio* simulation package (VASP)^27^ and Perdew–Burke–Ernzerhof (PBE) exchange-correlation function^28^ in canonical ensemble conditions. The equation of motion, which is governed by Newton’s second law, was integrated in the simulations using a Verlet algorithm with a time step of 1 fs. All MD simulations were performed for a total simulation time of 10 ps. The interaction between the ionic core and the valence electrons was described by the projector augmented wave method^29^, and the valence electrons were described using a plane wave basis up to an energy cut-off of 400 eV. The Brillouin zone was sampled at the Γ-point. The convergence criteria for the electronic structure and the geometry were 10^−5^ eV and 0.05 eV/A, respectively. A Fermi smearing function with a finite temperature width of 0.2 eV was applied to improve the convergence of states near the Fermi level.

The simulations were conducted using an optimised 4 × 4 × 10 Cu(111) slab. The bottom three layers of the slab were fixed during the simulations. To model the Cu–Zr interaction, four Zr atoms were placed on the three-fold hollow sites of the Cu(111) slab model surface. DFT-MD simulations were performed at 400, 1000, and 1380 K.

## Electronic supplementary material

Supplementary information
