# Supplementary material for: Minimising oxygen contamination through a liquid copper-aided group IV metal production process
Source: Sci Rep. 2018 Nov 26;8:17391. doi: 10.1038/s41598-018-35739-z (PMC6255757; doi:10.1038/s41598-018-35739-z)
Supplement: Supplementary file 1 — Supplementary information [file 41598_2018_35739_MOESM1_ESM.docx]

Supplementary Information for “Minimising oxygen contamination through a liquid copper-aided group IV metal production process”

BungUk Yoo^1^, YoungJun Lee^2^, Vladislav Ri^3^, SeongHun Lee^3^, Hayk Nersisyan^2^, HyunYou Kim^3^, JongHyeon Lee^1,2,3,*^, Nicholas Earner^4^, Alister MacDonald^4^

Author Address

^1^Department of Energy Science and Technology, Chungnam National University 99 Daehak-ro, 305-764, Republic of Korea.

^2^RASOM, Chungnam National University, 79 Daehak-ro, Yuseong-gu, Daejeon 305-764, Republic of Korea

^3^Department of Materials Science and Engineering, Chungnam National University, 99 Daehak-ro, 305-764, Republic of Korea

^4^Alkane Resources Ltd, 89 Burswood Road, Burswood, Western Australia 6100

Corresponding Author

Name: Jong-Hyeon Lee

Address: Department of Advanced Materials Engineering, Chungnam National University, 79 Daehak-ro, Yuseong-gu, 305-764 Daejeon, Korea

Tel: +82-42-821-6596

FAX: +82-42-822-5850

e-mail: jonglee@cnu.ac.kr.

Table and Figure Legends

Figure S1. Experimental apparatus for the electroreduction of ZrO_2_. (a) Solid Cu cathode system. (b) Liquid Cu cathode system. (c) Graphite anode.

Figure S2. Cyclic voltammograms and (b) reduction potentials of Ca^2+^ (CaO-free system) as a function of temperature.  (c) Polarisation behaviour during the electroreduction of ZrO_2_ in CaCl_2_ with 5 wt% CaO at 1380 K.

Figure S3. (a) Loading of the Ca and Cu granules in the Al_2_O_3_ crucible before melting.  (b) Surface morphology of the CaCu ingot after melting. (c) Bottom surface of the CaCu ingot. (d) Scanning electron micrograph and EDX analysis of the CaCu ingot bottom surface.

 Figure S4. Reduction behaviour of ZrO_2_ using CaCu alloy as a reducing agent.

Figure S5. CuZr ingots with various Zr contents produced by metallothermic reduction using CaCu. (a) 25 wt% Zr; ingot 1 and (b) 35 wt% Zr; ingot 2. These ingots were obtained by injection casting.

Figure S6. Micrographs and EDX analysis of CuZr ingots with various Zr contents produced by metallothermic reduction. (a) 25 wt% Zr and (b) 35 wt% Zr. Micrographs were obtained using the COMPO mode. (c) EDX compositional analysis of ingot 1 and 2.

Figure S7. XRD patterns of the CuZr ingots.

Figure S8. Electrorefining of Zr (a–e) Longitudinal and cross-sectional images of CuZr ingots under increasing applied electric charge. (f) Zirconium concentration changes according to depth profiling under different applied electric charges.

Table S1. Experimental details of the electroreduction of ZrO_2_ using the liquid copper cathode at 1380 K.

Table S2. Permissible (ASTM B349) and actual impurity levels in the low-Hf ZrO_2_ feedstock and the produced Zr ingot.

Table S3. EDX compositional analysis of CuZr.

Table S4. Metallothermic reduction conditions for preparing CuZr ingots


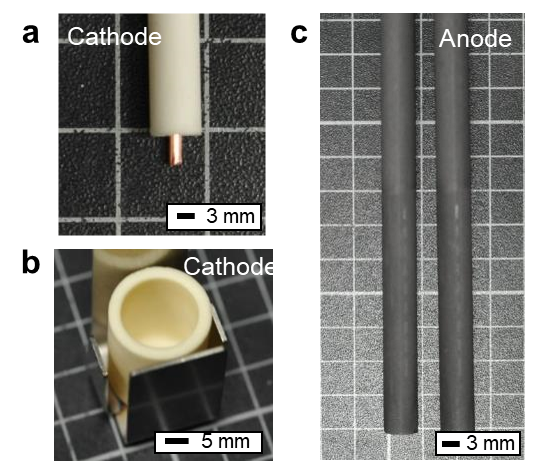


Figure S1. Experimental apparatus for the electroreduction of ZrO_2_. (a) Solid Cu cathode system. (b) Liquid Cu cathode system. (c) Graphite anode.


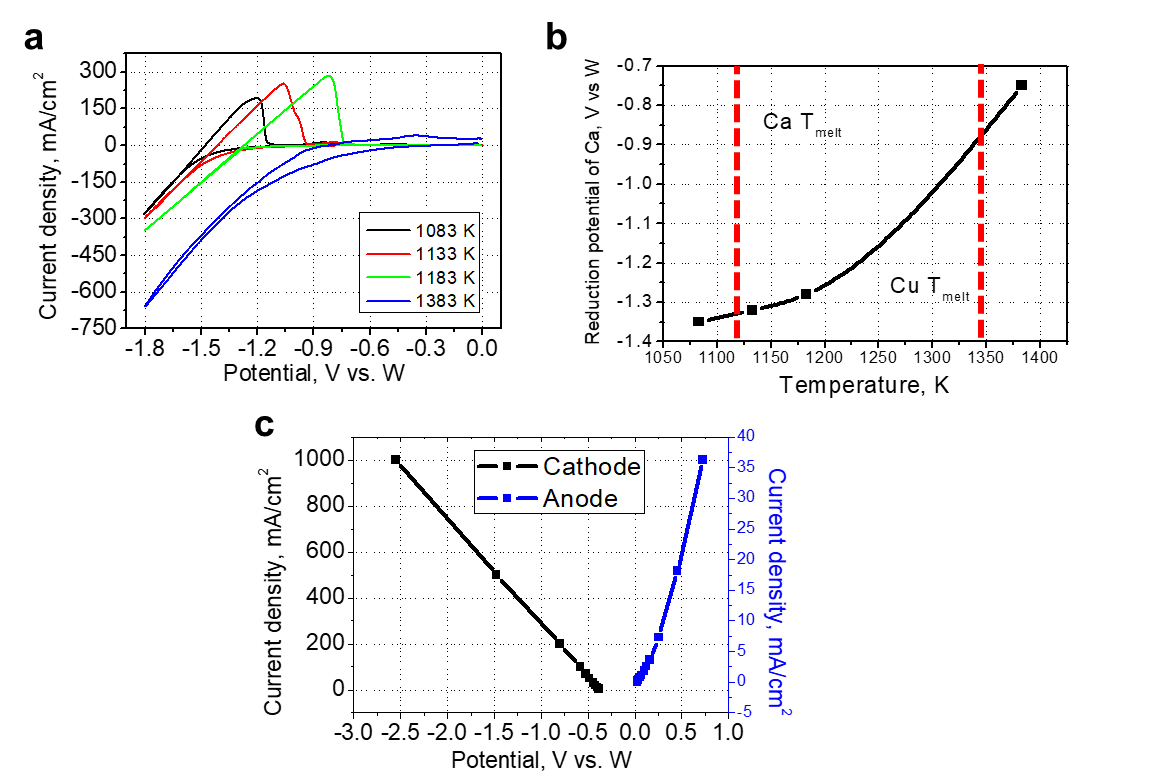
Figure S2. (a) Cyclic voltammograms and (b) reduction potentials of Ca^2+^ (CaO-free system) as a function of temperature. (c) Polarisation behaviour during the electroreduction of ZrO_2_ in CaCl_2_ with 5 wt% CaO at 1380 K.

Table S1. Experimental details of the electroreduction of ZrO_2_ using the liquid copper cathode at 1380 K.

| No | Cu, g | ZrO_2_, g | ZrO_2_/Cu ratio, wt. % | Applied current (density) | Time, hr |
| --- | --- | --- | --- | --- | --- |
| E1 | 10 | 0.62 | 6.2 | 1 A (500 mA/cm^2^) | 0.809 |
| E2 | 10 | 1.52 | 15.2 | 1 A (500 mA/cm^2^) | 1.984 |
| E3 | 10 | 4.01 | 40.1 | 1 A (500 mA/cm^2^) | 5.233 |
| E4 | 10 | 8.47 | 84.7 | 1 A (500 mA/cm^2^) | 11.054 |

Table S2. Permissible (ASTM B349) and actual impurity levels in the low-Hf ZrO_2_ feedstock and the produced Zr ingot.

| Element | Permissible Impurities,  Max (ASTM B349), ppm | Impurities of low Hf ZrO_2_ feedstock, ppm | Impurities of Zr ingot,  ppm |
| --- | --- | --- | --- |
| Al | 75 | 7.177 | <0.001 |
| B | 0.5 | - | <0.001 |
| C | 0.5 | - | <0.001 |
| Cd | 250 | - | <0.001 |
| Cl | 1300 | 397.187 | <0.001 |
| Cr | 200 | 4.018 | <0.001 |
| Co | 20 | - | <0.001 |
| Cu | 30 | 0.641 | 6.524 |
| Hf | 100 | 23.126 | 17.61 |
| Fe | 0 | 42.934 | 51.505 |
| Mg | 50 | 1.541 | <0.001 |
| Mo | 50 | - | <0.001 |
| Ni | 70 | 0.581 | 8.517 |
| N | 50 | - | 10 |
| O | 1400 | - | 891 |
| Si | 120 | 61.538 | 2.502 |
| Ti | 50 | - | <0.001 |
| W | 50 | <0.001 | <0.002 |
| U | 3.0 | - | 0.899 |
| Etc.(F) |  | <58.499 | 6.73(<0.001) |

Table S3. EDX compositional analysis of CuZr.

| E1 | | | |
| --- | --- | --- | --- |
| Element | Mapping, wt% | White, wt% | Gray, wt% |
| Al | 2.16 | 1.77 | 2.42 |
| Cu | 94.74 | 68.18 | 97.58 |
| Zr | 3.20 | 30.05 |  |
| Totals | 100 | 100 | 100 |
| E2 | | | |
| Element | Mapping, wt% | White, wt% | Gray, wt% |
| Al | 2.57 | 1.34 | 2.88 |
| Cu | 90.04 | 74.39 | 97.12 |
| Zr | 7.49 | 24.27 |  |
| Totals | 100 | 100 | 100 |
| E3 | | | |
| Element | Mapping, wt% | White, wt% | Gray, wt% |
| Al | 2.84 | 1.13 | 3.64 |
| Cu | 81.58 | 77.15 | 96.36 |
| Zr | 16.42 | 21.72 |  |
| Totals | 100 | 100 | 100 |
| E4 | | | |
| Element | Mapping, wt% | White, wt% | Gray, wt% |
| Al | 3.01 | 1.56 | 3.86 |
| Cu | 69.52 | 64.42 | 96.14 |
| Zr | 27.47 | 34.02 |  |
| Totals | 100 | 100 | 100 |

Metallothermic reduction using CaCu as a reducing agent

When a conductive metal crucible is used in the electroreduction process, CaCu intermetallic compounds cannot form because Ca is electrodeposited on the crucible outer surface. Consequently, ceramic and metal crucibles cannot be used in a electroreduction processes using a liquid Cu cathode. However, the CaCu-mediated metallothermic method can prevent the contamination of CuZr by any impurities from crucible materials. In addition, this method can be easily scaled up for commercial applications.

1. Preparation of the CaCu reducing agent

Materials and equipment used for the metallothermic reduction were the same as those used for the electroreduction. CaCl_2_ and ZrO_2_ were preheated at 623 K for 24 h to remove residual moisture. To prepare the CaCu alloy, Cu chips and Ca granules were melted in an Ar atmosphere glove box at 1173 K in an Al_2_O_3_ crucible for 2 h (Fig. S3a). The melting and solidification of CaCu were confirmed by breaking the crucible, which retained its surface integrity (Fig. S3b and S3c). The electron micrograph and EDX analysis of the CaCu ingot bottom surface showed that the molar ratio of Ca to Cu was nearly 1:1 as designed (Fig. S3d).


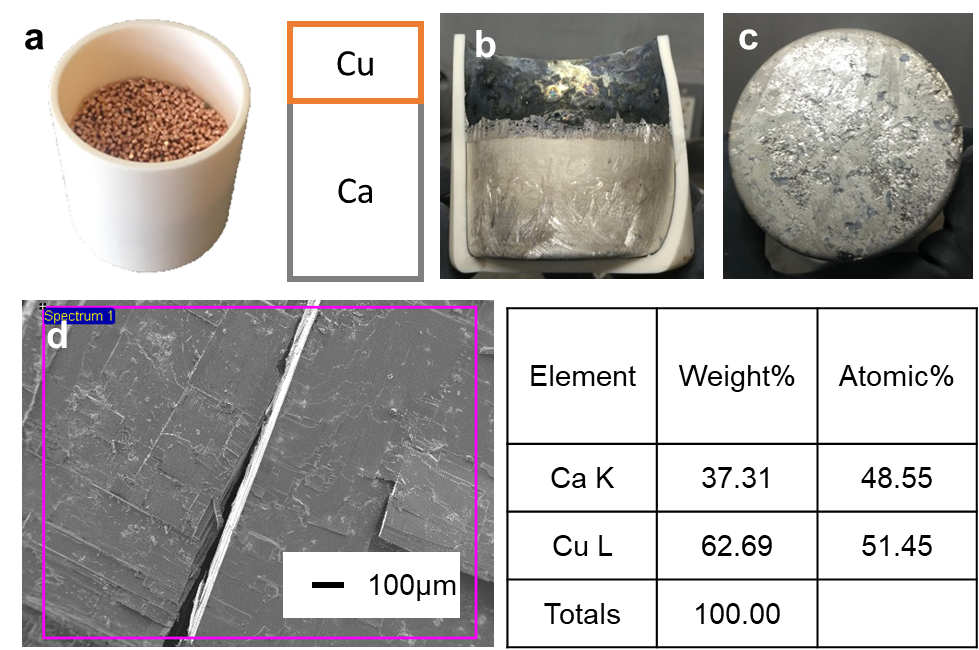


Figure S3. (a) Loading of the Ca and Cu granules in the Al_2_O_3_ crucible before melting. (b) Surface morphology of the CaCu ingot after melting. (c) Bottom surface of the CaCu ingot. (d) Scanning electron micrograph and EDX analysis of the CaCu ingot bottom surface.

1. Metallothermic reduction of low-Hf ZrO_2_ using CaCu

The reduction of ZrO_2_ into CuZr using a CaCu alloy is schematically shown in Fig. S4. Reaction conditions for preparing CuZr ingots 1 and 2 containing 25 and 35 wt% Zr, respectively, are listed in Table S4. In the process, CaCl_2_ and CaCu were melted in a Mo crucible (produced by Zhengzhou Shibo Nonferrous Metals Products Co., Ltd, China, 99.95%) with a 110-mm inner diameter placed in a glove box and heated to 1380 ± 10 K at a heating rate of 5 K/min using an electric furnace. Once the mixture was fully melted, ZrO_2_ was loaded into the Mo crucible. The metallothermic reaction occurred as ZrO_2_ was added. The mixture was allowed to react for 1 hour and stirred for 5 minutes every 20 minutes to sufficiently dissolve the produced CaO in the molten CaCl_2_. After stirring, the CaO-enriched CaCl_2_ was taken out of the crucible and replaced with fresh CaCl_2_ to dissolve the remaining CaO in the electrolyte by stirring in the same manner as above. As the residual CaO was dissolved, unreacted ZrO_2_ could be effectively reduced at the bottom of the crucible. The liquid CuZr formed at the bottom of the Mo crucible was recovered by quartz tube suction.


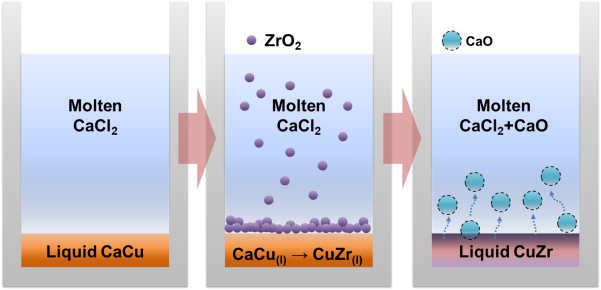


Figure S4. Reduction behaviour of ZrO_2_ using CaCu alloy as a reducing agent.

Table S4. Metallothermic reduction conditions for preparing CuZr ingots

|  | Condition 1 (ingot 1) | Condition 2 (ingot 2) |
| --- | --- | --- |
| CaCl_2_ | 3.5 kg | 3.5 kg |
| CaCu | 1.3 kg (24 wt% Ca) | 1.3 kg (34 wt% Ca) |
| ZrO_2_ | 435 g | 588 g |
| Temperature | 1383 ± 10 K | 1383 ± 10 K |

1. Characteristics of CuZr ingots prepared by the CaCu-mediated metallothermic reduction

The metallothermic reaction between CaCu and ZrO_2_ gives CuZr as:

Ca_2x_Cu + xZrO_2_ = CuZr_x_ + 2xCaO (S1)

Based on this equation, the anticipated Zr concentrations amounted to 25 and 35 wt% in the product for the conditions listed in Table S4. Also, CuZr was expected to exist as a liquid phase at 1380 ± 10 K. Therefore, after the reaction was completed, a liquid metal mixture recoverable by suction was present at the bottom of the crucible. The recovered metal was solidified by injection casting in the Ar atmosphere glove box (Fig. S5). The recovered ingot was cross-sectionally cut and polished for SEM analysis in COMPO mode (Fig. S6). The EDX analysis of CuZr ingots 1 and 2 led to Zr contents of 25 (Fig. S6a) and 35 wt% (Fig. S6b), consistent with the predictions. As a result of the point analysis, point 2 was identified as a structure with a high Zr content, and the point 1 was a CuZr-type structure with a high Cu content. Unlike in the electroreduction process, no Al and Mo contamination from crucible materials were observed. Black dots present in the middle of the ingot were pores (Fig. S6a), which may result from fast cooling. As predicted, CuZr ingot 1 contained 25 wt% Zr, suggesting that Zr replaced all of the Ca in CaCu. In addition, XRD results showed that the ingot consisted of CuZr, CuZr_2_, and Cu_5_Zr alloys as well as a small portion of Cu (Fig. S7). It is postulated that the higher the Zr content of the CuZr ingot, the more the pure Cu region disappeared and was converted into CuZr_2_^1^. Therefore, it is possible to produce nuclear-grade Zr from these CuZr ingots by electrorefining.

1 Wang, N., Li, C., Du, Z., Wang, F. & Zhang, W. The thermodynamic re-assessment of the Cu–Zr system. *Calphad* **30**, 461-469 (2006).


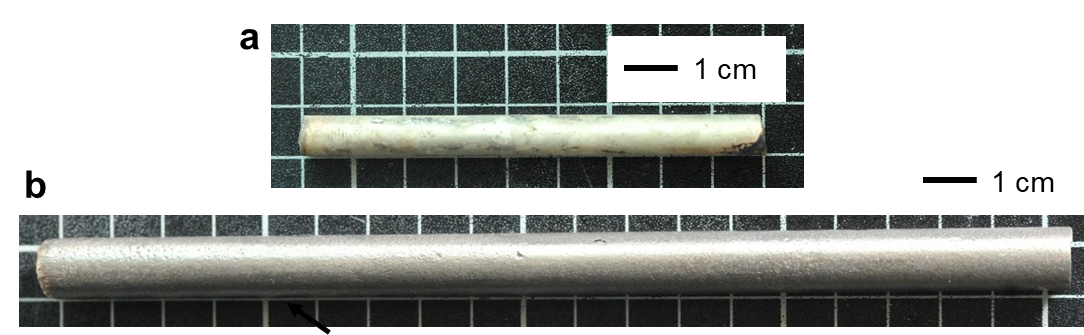


Figure S5. CuZr ingots with various Zr contents produced by metallothermic reduction using CaCu. (a) 25 wt% Zr; ingot 1 and (b) 35 wt% Zr; ingot 2. These ingots were obtained by injection casting.


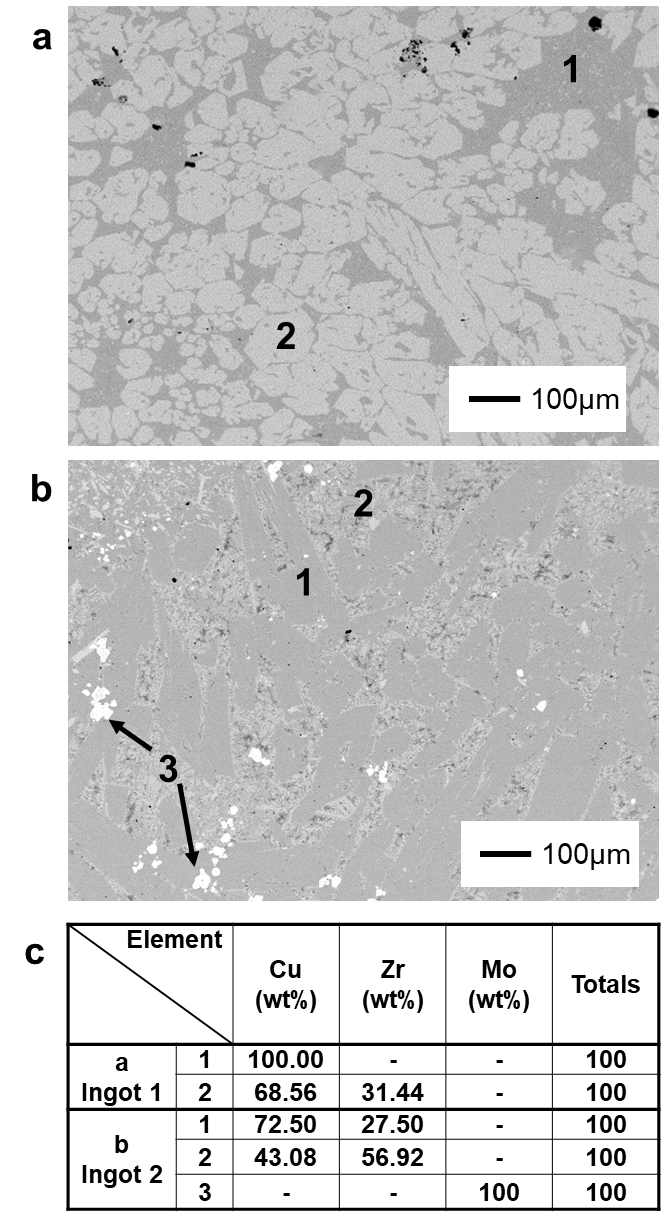


Figure S6. Micrographs and EDX analysis of CuZr ingots with various Zr contents produced by metallothermic reduction. (a) 25 wt% Zr and (b) 35 wt% Zr. Micrographs were obtained using the COMPO mode. (c) EDX compositional analysis of ingot 1 and 2.

Figure S7. XRD patterns of the CuZr ingots.

Anodic dissolution of Zr by electrorefining

To confirm the Zr elution of the anode, electric charges of 3.64, 5.09, 12.41, and 48.41 Ah were applied to the Cu–35 wt% Zr anode. Current densities amounted to 55 mA/cm^2^ at the anode and 100 mA/cm^2^ at the cathode under each applied electric charge condition. Figures S8a–S8e show cross-sectional photographs of the anode according to the applied electric charge. The changes in Zr concentration in the CuZr ingot were analysed by EDX according to the distance from the anode surface for different applied electric charge conditions (Fig. S8f). As the electrorefining process proceeded, Zr dissolved from the anode surface while the anode itself gradually became concentrated in the copper. As the applied amount of current increased, the copper colour expanded from the surface to the centre until it covered the entire area. However, even when the entire region showed a copper colour, about 3 wt% of Zr remained (Fig. S8f). This is because the Zr was eluted from the ingot, and the electrolyte occupied the vacancy, as evidenced by the coexistence of Ba and F elements in the EDX analysis. It can be concluded that most of the Zr was eluted from the CuZr ingot, which contained under the detection limit according to the EDX point analysis. Most of the Zr was recovered at an electric charge of 48.41 Ah and the current efficiency of the anode corresponded to 182% of the theoretical electrochemical equivalent, so that the Zr could finally be recovered. However, as the electrorefining process proceeded, the CuZr anode became unstable and partly desorbed. Therefore, it is necessary to conduct a refining experiment using a low-Zr anode (< 35 wt% Zr) to optimise the Zr content.


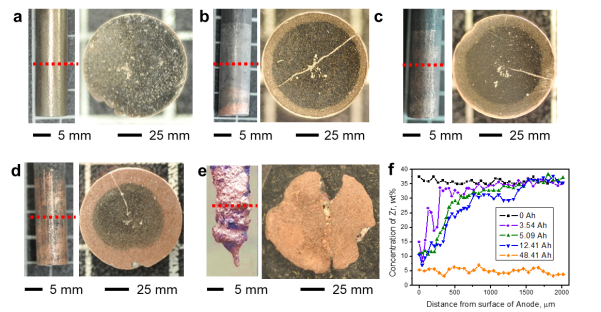


Figure S8. Electrorefining of Zr (a–e) Longitudinal and cross-sectional images of CuZr ingots under increasing applied electric charge. (f) Zirconium concentration changes according to depth profiling under different applied electric charges. The red dotted line indicates the cutting site.
